# AHI1 gene mutation in a consanguineous Iranian family affected by Joubert syndrome: A case report

**DOI:** 10.1002/ccr3.5002

**Published:** 2021-10-23

**Authors:** Mostafa Neissi, Hadideh Mabudi, Javad Mohammadi‐Asl

**Affiliations:** ^1^ Department of Genetics Khuzestan Science and Research Branch Islamic Azad University Ahvaz Iran; ^2^ Department of Genetics Ahvaz Branch Islamic Azad University Ahvaz Iran; ^3^ Department of Medical Genetics School of Medicine Ahvaz Jundishapur University of Medical Sciences Ahvaz Iran

**Keywords:** AHI1 gene, Joubert syndrome, mutation

## Abstract

This point of detected mutation could be considered as a novel mutational hotspot point that carried in patient ancestors. Moreover, the obtained results and family history suggest a precise genetic consulting and molecular prenatal evaluation for suspect individuals with a family history of mental and physical abnormalities.

## Graphical abstract

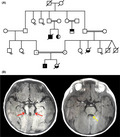

## 1 INTRODUCTION

The first description of a rare new disease named Joubert syndrome (JBTS) goes back to 1968 when it was discovered in a family whose four siblings suffered from a set of mental and physical disorders, including the appearance of the cerebellar vermis, hyperpnoea, disability in eye movements, ataxia, and intellectual abnormality.^1^ Then, JBTS was more introduced as an autosomal‐recessive inherited disorder characterized by brain abnormalities, delayed growth, the molar tooth sign, and unusual organ malfunctions such as ocular motor apraxia and respiratory disorders.^2^, ^3^ JBTS is currently diagnosed by evidence of the molar tooth sign (MTS) in the patient's brain. Brain magnetic resonance imaging (MRI) usually demonstrates related CNS defects affected mainly by the tectum and midbrain.^2^ Although the MTS is a prominent characteristic feature of JBTS, it has been recognized in various disorders titled Joubert syndrome‐related disorders (JSRDs), demonstrating the JBTS neurological aspects associate with organ involvement that it can be related with more central nervous system (CNS) deformities.^4^ JBTS is a heterogeneous illness in which mutations in more than 35 ciliopathy‐related genes have been associated with the disease manifestations.^5^

We reported a case showing typical signs of JBTS with hypotonia, mental retardation, poor language skill, walking problem, and the hallmark of MTS on MRI. In this case, we performed whole‐exome sequencing followed by a targeted sequencing approach to identify the underlying genetic defect.

### 1.1 Sampling and DNA extraction

The patient family for this study was referred to the Noor Gene Genetic Lab, Ahvaz, Iran. Whole blood samples from the patient (son of the family) and his parents were informed consent. According to the standard protocol, DNA was extracted from the buffy coat using FAVORGEN kit (Biotech Corp, Cat. No.: FABGK 001, Taiwan). Written informed consent was obtained from all participants, and the study was carried out according to the Ethics Committee of Iran's Ministry of Health and Medical Education guidelines.

### 1.2 Exome sequencing and mutation analysis

Whole‐exome sequencing and targeted Sanger sequencing on exons and intron‐exon boundaries of the AHI1 gene were performed using ABI‐3130 XL (USA) for the affected son and his parents. A standard protocol to amplify the polymerase chain reaction (PCR) condition was used. The sequences, region of mutation, and homozygosity (ROHs) were analyzed using UGENE software.

### 1.3 Clinical report

**FIGURE 1:**
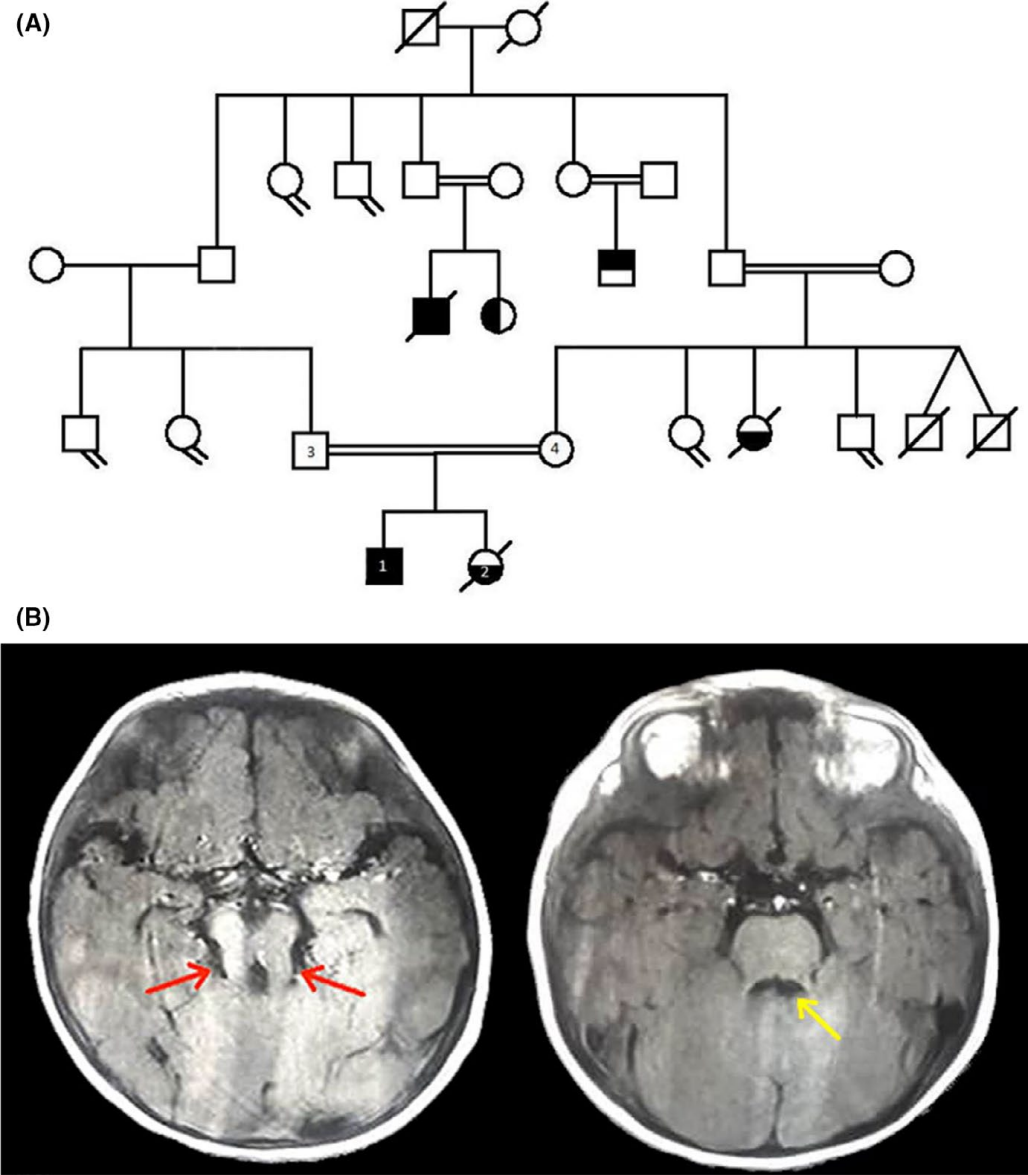
A. Pedigree of the studied family. Patient 1 was a 4‐year‐old affected boy in the presented study and number 2 was 4‐month dead girl. The parents of the affected son (3,4) are first cousins. B. T2 weighted the molar tooth sign of the midbrain (red arrows) and the bat‐wing configuration of the 4th ventricle (yellow arrow)

The patient is a 4‐year‐old boy (Figure 1) referred to medical genetics for visual impairment and physical weakness. He had hypotonia, psychomotor delay, and impaired language skills. He also cannot control voluntary muscle movements (ataxia), such as walking, picking things up, and speaking, and finally, a brain MRI indicating MTS, but his parents are healthy people.

### 1.4 Molecular analysis

**FIGURE 2:**
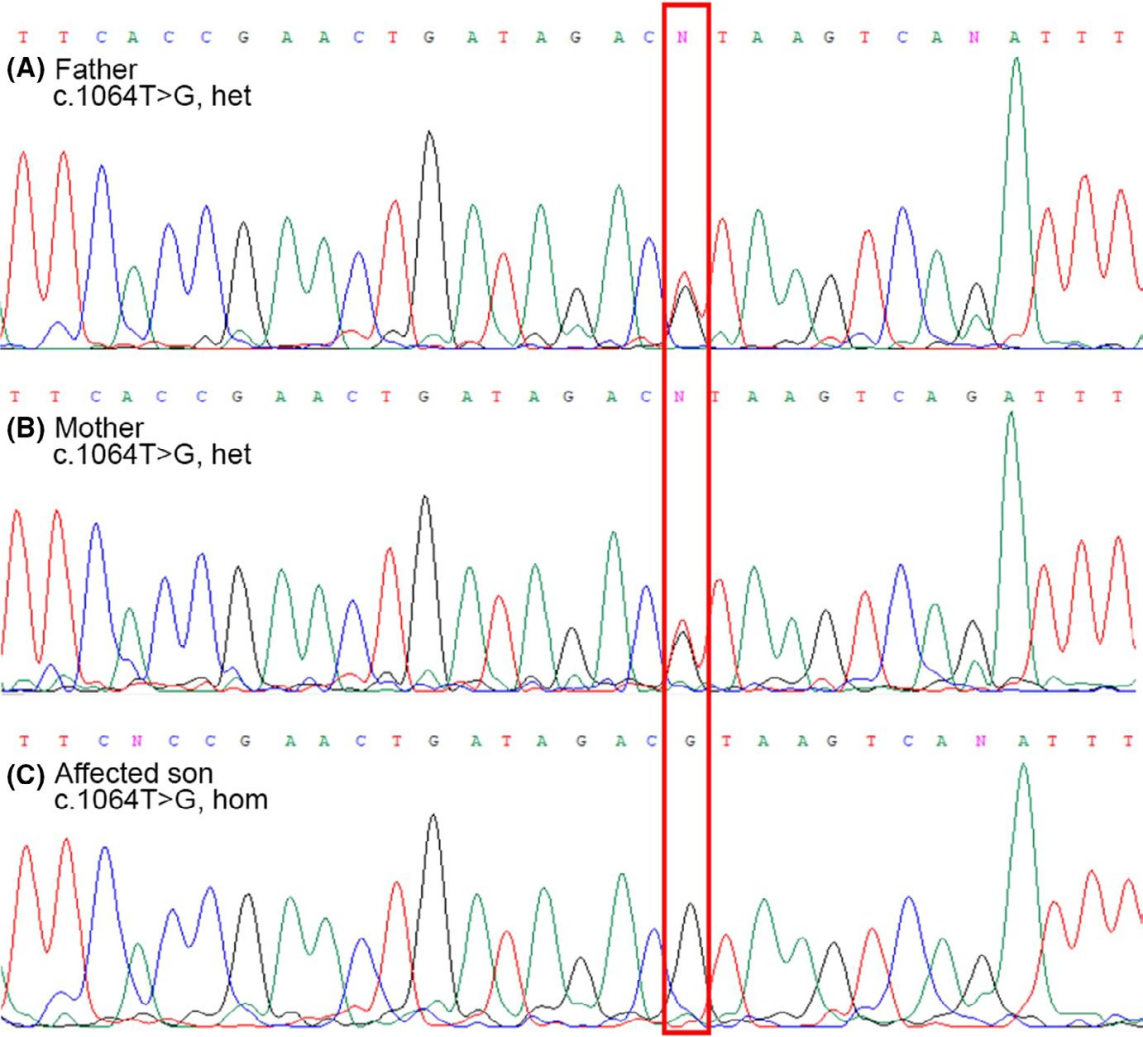
Sequencing data analysis. The patient carries a homozygous mutation (p.L355R: c.1064 T>G), causing a missense mutation (C: affected son). Parents are heterozygous for the detected mutation (A, B)

Whole‐exome sequencing and direct Sanger sequencing were conducted on DNA lymphocytes separated from peripheral blood samples of the family. Results showed a single homozygote mutation in the affected son and heterozygous in parents. We investigated the compatibility of the detected homozygous mutation with known JBTS loci and confirmed that the homozygous mutation on chromosome 6q encoded Abelson helper integration site 1(AHI1). Sanger sequencing of coding exons showed that this single mutation (c.1064 T>G; p.L355R), located in exon 7 (NM_001134830), causing a missense mutation, predicting an alteration in codon translation, and finally change coding protein (Leucine converted to Arginine). The transformation was homozygous in a patient (Figure 2) and heterozygous in his parents. Nonspecific bands or contamination was not detected in the control specimen.

## 2 DISCUSSION

JBTS is a scarce and inherited autosomal‐recessive ailment with no accurate epidemiological reports. Although some estimations declared the mean prevalence of JBTS is about 1 per 90,000 live births,^6^, ^7^ this variant of uncertain significance is not reported yet. Some investigators believe that the number of patients is beyond this estimation, and that is why the molar tooth sign is still unfamiliar in patient clinical evaluation.^7^, ^8^, ^9^ So far, reports on genetic defects of JBTS indicated that about 35 ciliopathy‐associated gene mutations are involved in disease pathogenesis, including CEP290, TCTN1, CC2D2A, TCTN3, NPHP1, INPP5E, TCTN2, and AHI1 genes.^5^

Hence, in this study, we investigated the disease‐causing mutation in a 4‐year‐old affected boy, who referred to medical genetics for visual impairment and physical weakness. The patient suffered from hypotonia, psychomotor delay, and impaired language skills. In addition, this family reported death of a 4‐month daughter with a suspicious history of JBTS, but parents are healthy people. The whole‐exome sequencing technique was used to identify the impaired gene in the family. In the next step, we conducted direct Sanger sequencing of the AHI1 gene to detect the exact location of gene mutation. The homozygous p.L355R: c.1064 T>G (exon 7 for NM_001134830) mutation was detected in the patient (4‐year‐old boy), followed by heterozygous mutations in his consanguine parents resulted in inhomogeneity in their child.

AHI1 is the product of the AHI1 gene, jouberin protein, a positive regulator of the Wnt signaling pathway, and promotes β‐catenin nuclear translocation.^10^ Since most detected mutations in the AHI1 gene are truncating, and there are few reported missense mutations,^7^, ^11^, ^12^ the finding of our study added a new missense mutation to exiting knowledge. We proposed that this novel missense mutation encodes an impaired protein that probably defects in function or stability, resulting in JBTS clinical manifestations.

It was demonstrated that AHI1 mutations are more prevalent in the Arab population.^13^ Since considerable Arab families live in Khuzestan province, even if this mutation previously appeared in a homogeneity state, it has not been discovered. The probable death of affected individuals of lower ages, as in this family, can be an explanation for this. Therefore, it can be interpreted that this episode is due to a founder effect and most mutations in JBTS patients. However, there is another hypothesis that this mutation just has occurred during the development of a mutational hotspot.

On the contrary, AHI1 mutations are more prevalent among the disease‐causing mutation of specific forms of JSRD with different frequencies.^12^ Kroeset al. reported that 16% of JBTS patients in their study carried AHI1 mutations.^11^ In the report published from a large cohort study by Akella Radha Rama Devi et al., AHI1 gene mutation was detected in 2 (5.4%) patients out of 59 JBTS patients.^5^ In a study, Enza Maria Valente et al. evaluated the families with pure JBTS or JBTS with retinal and/or central nervous system defects for AHI1 mutations. Their results showed a frequency of 7.3% and with only one missense mutation in the AHI1 gene.^12^ They concluded that this form of JBTS involving AHI1 mutation is accompanied by retinal involvement and childhood blindness.^12^ In another investigation, Kroes et al. revealed that AHI1 mutation correlates with retinal abnormality^11^ and this finding is consistent with our result.

It was demonstrated that patients with AHI1 mutations are highly related with pure JBTS or JBTS with retinal impairments with different age at diagnosis, progression, and intensity^7^, ^14^, ^15^, ^16^; also, AHI1 mutations recognized as one of the most prevalent gene defects in this subgroup of JBTS with frequency nearly 20%.^7^

Studies showed that renal dysfunctions consistent with NPHP gene mutation have also been observed in JBTS patients carrying AHI1 mutations.^6^, ^11^ Nevertheless, in the affected case presented in this study, we did not observe any renal or breathing abnormalities. Our finding revealed that exome sequencing in consanguine parents is a valuable tool to detect probable or novel mutations causing JBTS, especially in suspect individuals with a family history of retardation and physical abnormalities.

## 3 CONCLUSION

The present study detected a case of JBTS with a rare homozygote mutation (p.L355R: c.1064 T>G) in the AHI1 gene in the son of a family from heterozygote and carrier parents using the Sanger sequencing technique. It can be concluded that this point of detected mutation is a rare mutational hotspot point that carried in patient ancestors. Moreover, the obtained results and family history suggest considering this AHI1 gene mutation in the genetic test platform of JBTS cases.

## CONFLICT OF INTEREST

The authors declare no conflict of interest.

## AUTHOR CONTRIBUTIONS

MN contributed to the discussion and edited the manuscript. MN and JMA analyzed and interpreted the data. HM wrote the manuscript. All authors have read and approved the final manuscript.

## CONSENT

Written informed consent was obtained from the family for this publication.

## Data Availability

The data that support the findings of this study are available from the corresponding author upon reasonable request.
